# A conceptual design of circular adaptive façade module for reuse

**DOI:** 10.1038/s41598-023-47593-9

**Published:** 2023-11-23

**Authors:** Dalia Niazy, Esraa A. Metwally, Mostafa Rifat, Mohammed Ibrahim Awad, Ahmed Elsabbagh

**Affiliations:** 1 Architecture Department, Faculty of Engineering, Ain Shams University, 1 El Sarayat St., Abbasseya, El Weili, Cairo, 11517 Egypt; 2 Mechatronics Engineering Department, Faculty of Engineering, Ain Shams University, Cairo, Egypt; 3 Production Design and Engineering Department, Faculty of Engineering, Ain Shams University, Cairo, Egypt

**Keywords:** Climate sciences, Energy science and technology, Engineering

## Abstract

Climate change has an impact on the ecosystem, and subsequently, it affects the built environment. Building envelope has a vital role in controlling the integration between indoor and outdoor environmental quality. The responsivity of the façade has proven its efficiency in optimizing the global energy performance of buildings. Adaptive façades are multifunctional reconciling envelope dynamic systems that improve sustainability with the purpose of utilizing environmental parameters. This paper tackles the research gap in integrating façades circularity, adaptive envelopes, and design for disassembly. The research investigates the merge between biodegradability, circularity of adaptive façades components, and interior space micro-climate control for energy efficiency. This paper presents a proof of concept for a circular adaptive façade during two phases in its life cycle: operation and reuse phases. A scientific quantitative method took place which is based on a hybrid method; computational simulation, smart control, and an up-scale model. Adaptability is investigated through the façade life cycle from design to disassembly instead of demolition and consequent waste production, by exploiting sustainable materials. As a result, an empirical prototype is constructed. The prototype provides 3 levels of adaptability across the design, operation, and disassembly for reuse. Subsequently, this work proposes an up-scale physical model that can help in mitigating the climate change effects.

## Introduction

The buildings’ energy performance impacts global energy consumption and emissions; thus, it has a significant impact while implementing the sustainable development goals (SDGs) of climatic actions and city development^1^. Consequently, urgent actions by the government and NGOs are needed the adapt to these new conditions that enable people to judge, act, and mitigate the consequences. Energy consumption and pollution are significant causes of climate change impacts and global warming. Many studies demonstrated that buildings are responsible for around 40% of global energy consumption^2^. This impact contributes to the climate change issues which affect our built environment^3^. The European Commission admitted that we need innovative design trends and professional efforts to focus on improving energy savings^4^. The European Union (EU) requires Member States to develop long-term national plans to encourage energy efficiency and reduction of CO_2_ emissions by between 80 and 85% compared to 1990 Directive UE (2018/844). According to the latest Egyptian electricity holding company annual report^5^, 58.3% of Egypt’s electricity consumption is accounted for by the *building* sector. Thus, the building’s energy consumption today is of critical consideration and is included in Egypt’s National Climatic Change Strategy (NCCS) 2050^6^.

Building envelopes plays a critical role in controlling the indoor climate. The concepts of adaptive façades are based on a variety of materials, components, and control systems to provide viable solutions for reducing energy consumption and ensuring comfort improvements^7^. Adaptive skin façades have been proven through several research for their efficient impact to respond to the changing climate and reduce electromechanical loads^8–11^. In addition, the current development of digital controls, networks, and connectivity technologies helps to enhance the human experience and reduce building energy consumption through smart control. Subsequently, an assessment tool^12^ was developed to evaluate the implementation levels and the impact of networking applications in buildings, within four levels as users’ preferences and activities in each space, space level that assesses tasks and space parameters, building control and operation levels including maintenance, data and facility management and the relations between the building and the city. Interactive adaptive façades evolved through research work from basic movable louvers^13^ to advanced global systems – which adapt to weather and climatic changes – that utilize energy harvesting^14–16^. In recent eras, computational advances resulted in intelligent façades^17–20^ and biomimetic responsive ones^15,16,21^ allowing advanced control strategies and responsive facade design optimization.

There is a strong correlation between the design of a building envelope, sustainability, construction process, and waste production^22^. Biodegradable materials offer lower energy consumption compared to others, which can reduce energy consumption and CO2 production by 20%^23^. Fabrics as biodegradable construction materials have different advantages for architectural buildings. The translucency of fabric structures helps to save lighting and energy costs, through adaptation, transformation, interaction techniques, and recycling^24–26^. Depending on the indoor environment functions the natural lighting can improve the workspace quality and users’ productivity. Not only for the design flexibility, permanent or transportable, cost-effectiveness, transparency, and climate control but also from users’ perspective, it could attain the aesthetical needs of occupants. While the economic needs could be settled by adaptation and mobility. In ref.^27–32^ they discussed the adaptive solar façade solutions for designing a modular highly integrated dynamic building façade using various methods and materials.

A gap is deduced from the literature that design for circularity of adaptive/interactive façades was not when the building is demolished and disassembled. This paper presents a proof of concept for circular adaptive while taking into consideration the biodegradability, circularity, and adaptive facades concepts. The work presented is part of a research project that focuses on developing a circular biodegradable adaptive façade system module for enhancing buildings’ energy efficiency and performance in responding to climate changes in Egypt. This can allow users to easily adapt specific parameters of the space corresponding to the climate changes, which are the occupants’ visual and thermal comfort, and the lighting consumption. The project builds upon the authors’ previous work on biodegradable responsive envelopes^33^. The significance of this proposed circular prototype is to introduce this idea to the market which could be further developed to attract business sponsors and produce a sustainable local product. As it can effectively be replicated in different climate zones in Egypt. The key operational objectives of this research are as follows:

- Optimizing façade life cycle. Through promoting a different design-to-disassembly approach to minimize building waste.
- Ensuring the interactions between buildings, climate change, and subsequent potential effects on building occupants, energy efficiency, and the built environment.
- Minimizing the electromechanical loads.

**Figure 1:**
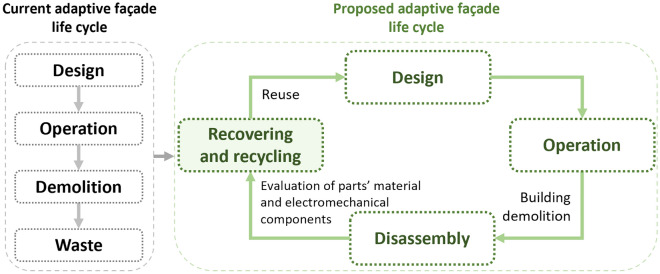
The research gap illustration and the shift for circularity in the design approach of adaptive façades.

The life cycle of the façades will be optimized as shown in Fig. 1 through the four R’s concept application to adaptive façades, which is reuse, recover, recycle, and reduce. Material reduction and reuse plans are implemented in the design phase. Recycling and recovering are implemented in the evaluation phase preparing for reuse. The interior space energy consumption will be controlled by investigating the optimum material and motion assembly selection that can affect responsivity for the changing external building environment. To address these points, the following design objectives were implemented:

- Constructing a responsive biodegradable façade prototype fitting for adaptive reuse to mitigate the effects of climate change.
- Utilizing local biodegradable materials in construction.
- Balancing efficient utilization of biodegradable and non-biodegradable materials for optimized circular façade system performance and durability.
- Minimizing the number of actuators with an advanced assembly for different louvers’ motion.

A scientific quantitative method through hybrid testing is used, which combines a small-scale real-life environment with a virtual simulated tool. A four-phase approach is used as follows; computational modeling and simulation phase, constructing the model phase, software programming with hardware (control) phase, and finally the testing phase.

This paper aims to develop an adaptive façade prototype that considers the circularity of façade elements when a building is demolished and disassembled, by using local materials and minimizing the climate change impact on buildings. The work Sect. **“**Literature review**”** presents the state of art and research of adaptive kinetic façade structures by analyzing each approach of different cases, resulting in a deduced workflow for the proposed biodegradable façade of this research and the idea behind the structure modeling. In Sect. “Methodology, material, design and methods”, the methodology through materials and methods for the design and fabrication is presented. In Sect. “Results and discussion”, the simulation results, and the empirical study of this research through fabrication are presented along with a discussion. Finally, Sect. “Conclusion and future work” presents the conclusion and future work of this project.

## Literature review

### Climate change, building envelope, and inhabitants’ comfort

By far, building envelopes are expected to make a bigger contribution to ensure indoor comfort abandoned from the extreme conditions caused by climate change, which will provide humans with adequate health and well-being conditions during building operations. That is related to some factors such as thermal comfort and visual exchange between indoors and outdoors. So, more efforts should be made to propose different dynamic solutions that can contribute to passively controlling the radiation exchanges to reduce the energy usage in our buildings. Accordingly, the proposed façade elements must be designed not only to be able to respond to different variables and new climatic conditions but also to be adaptive to ensure various efficiency and functionality levels^34^.

Climate-adaptive building envelopes should provide significant effects to improve functional requirements of the envelope in terms of heating, air, and water vapor flow, solar radiation as well as occupants’ visual and thermal comfort. One of the biggest challenges is to develop a universal envelope adaptive design solution that has the capacity for adaptation in different circumstances with diverse conditions^35^. In ref.^36^, they proposed the idea of promoting the implementation of multifunctional façades in building envelopes. Such as different blocking angles for a blind regulating shade in different sun latitudes and seasons of the year by using some technologies such as sensors, actuators, and tags. In ref.^37^, the importance of human comfort was discussed, and indoor environmental quality control was analyzed due to the drastic change in climatic change and global warming. Focusing on how to study the factors that govern indoor human comfort within space. The research highlighted a research need to predict human comfort to maintain the optimum indoor environmental quality. In ref.^38^, it demonstrated the diversification of theoretical methods in energy research dealing with climate change impacts. Through exploring the issues of mitigation strategies, behavioral psychology, and technology prospects.

Bionic structures and physical principles served as the basis for the algorithms used to calculate geometry^39^. In ref.^18^ a linear optimization process associated with a comparison method was applied to find the best sets of the louver by investigating the reflectivity range, number of façade elements, window-to-wall ratio, rotation angle, and depth, which were used as variables to improve visual comfort. In addition^19^, a kinetic hexagonal pattern was evaluated in comparison to a fixed conventional south-oriented façade. The results indicated that the proposed system could improve daylight by 50% in summer and spring, and 20% in wintertime. Moving to^20^, they provided a set of louvers that could respond automatically to the sun’s position. The louvers set could parametrically reflect the sun rays to the ceiling to provide more visual comfort quantity and daylight distribution. A predefined sky condition and sun path diagrams were used to set the louver configuration and control strategy while focusing on a south-oriented façade under a clear sky at critical dates and times of the year. The adaptive solar façade module in^40^ utilized material properties of a specific part of the adaptive façade system for soft actuation. The façade consisted of solar panels as shade and a soft pneumatic controller. The controller gained its motion energy by partial consumption of the electricity produced by the solar panel. Thus, providing another non-renewable electric energy consumption elimination.

### Biodegradable materials in construction

Biodegradable materials are becoming more and more popular in several industries, they offer lower energy consumption compared to other materials. A good example of biodegradable materials in construction is using fabrics in architectural buildings^41,42^. Depending on the indoor environment functions, the natural indirect lighting can improve the workspace quality and users’ productivity. Fabrics are compatible with building materials and are flexible to manufacture. There are different types of fabrics used in Architecture so far such as polyester, polyethylene, fiberglass, nylon, Teflon, PTFE, silicone, and acrylic. In Al Bahar Towers in Abu Dhabi, the adaptive façade is controlled by sensors and actuators during daylight hours. The origami panels are covered by a polytetrafluoroethylene (PTFE) coated fiber mesh, which reduces the G-value of the façade by more than 50% as compared to a glazed envelope^25^. In ref.^26^, they used TFP (Tailored Fiber Placement) textile that could perform processes to architectural applications and to adapt design methodologies accordingly.

### Identification of the research-based criteria

**Figure 2:**
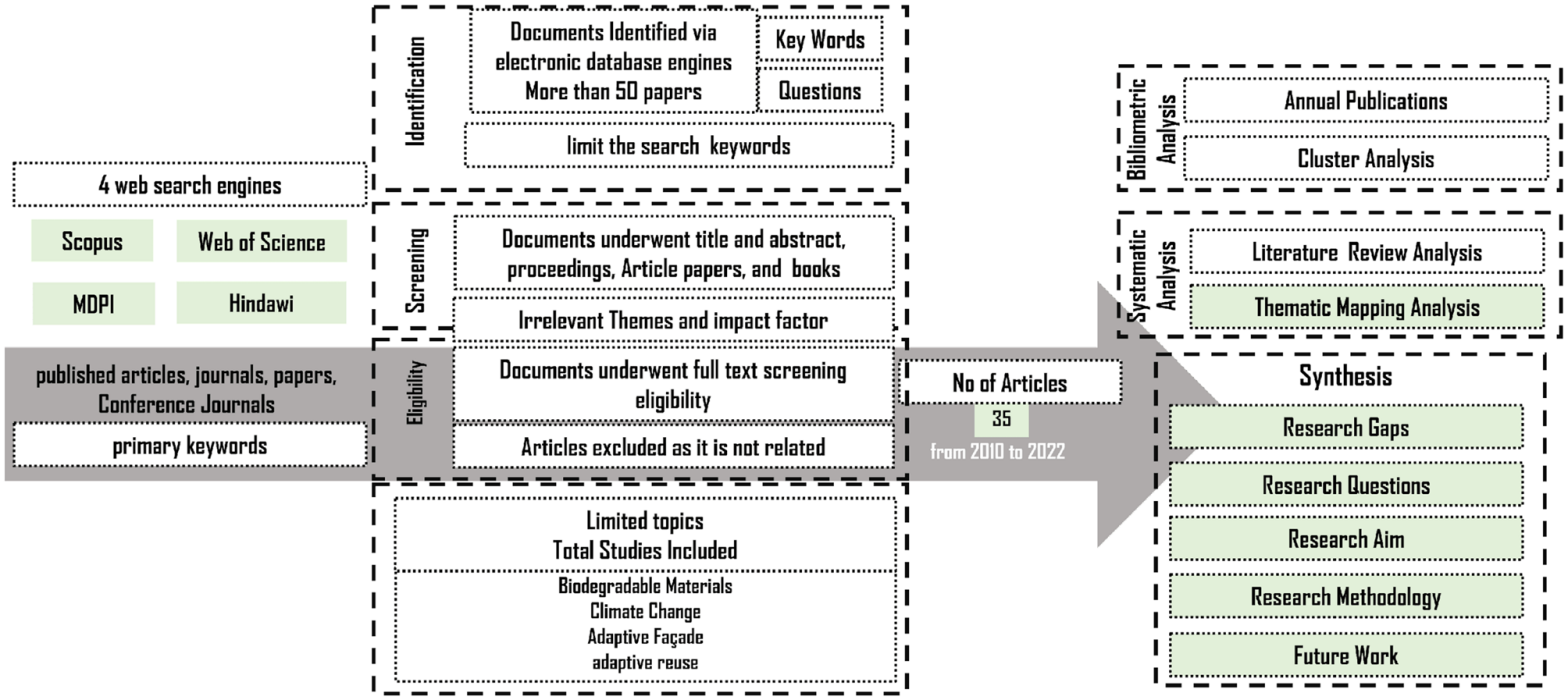
Flowchart of selecting research-based criteria/sub criteria and analysis by using different research web engines, source by the authors.

The main key attributes defined from the literature for the proposed module prototype are illustrated in Fig. 2. It demonstrates the different façade criteria parameters, sub-criteria parameters, and the different key attributes deduced from the literature. Based on 4 web search engines from Scopus, Web of Science, MDPI, and Hindawi from 2010 to 2022, we could collect the data used in this research. Documents were identified by selecting the main keywords of this research as adaptive Façade, adaptive reuse, biodegradable Materials, circularity, and climate change. Through further screening and eligibility steps, 35 publications are selected as most relevant. The next step is to build the bibliometric and systematic analysis to identify the related research gaps, current state of research, methodology, and tools.

Classification of literature is done according to mechanism and motion, material, design, mechanism, and intelligence. In addition, the aim and methodology of each literature and how to address the significance of each idea. Table 1 classifies the selected literature of this study to set the proposed framework prototype, based on the previous literature discussed above.

**Table 1:** A review of the selected literature and their classification.

Ref.	Criteria	Aim	Results
^38^	Mechanism and Motion	Presents the adaptive solar façade (ASF), a modular highly integrated dynamic building façade. For a more comfortable thermal and visual environment that fulfills the requirements of the user for shading, temperature, and light levels. In addition, the power should be generated through the PV cells	The shading panel consists of a 0.8 mm thick aluminum substrate for the thin-film PV module, the soft-pneumatic actuator is made from elastic materials, e.g. silicone rubber, and energized using compressed air, the cantilever, and the supporting frame and cable net. It weighs 800 g with design considerations for the actuator, the cantilever, and the cable net
^27^	Mechanism and intelligence	The building seeks energy self-sufficiency, so it has an ecological roof with photovoltaic panels that can produce 29.000kWh yearly, saving up to 18.8 tons of CO_2_ emissions to the atmosphere. Using indoor sensors to control light and temperature depending on occupation	The patterns are clad in ETFE cushions with two different patents: the ‘cloud’ system is filled with a combination of air and nitrogen that uses the density of the air as a sun filter in the southwest In addition, the ‘diaphragm’ system, with three ETFE layers forms in constant pressure and variable air circulation between chambers in the southeast. The exterior layer is transparent, whereas the middle and inner one have a pattern that forms an opaque layer
^28^	Material and Motion	self-adaptive membrane utilizes kinetic joints responsive to solar radiation	It is the result of their research into the space memory alloy nitinol, nickel, and titanium which was utilized to develop a passive kinetic motor capable of adapting in response to environmental cues
^29^	Material	Adaptive façade systems as soft robotics in architecture	The project investigates the potential of adaptive soft robotics which addresses many issues in energy efficiency as well as user comfort. The project integrates the fabrication of concept prototypes with robotics as well as their testing
^30^	Design	The air and wind would be channeled into the building and filtered to provide clean air and natural air conditioning	The active skin would be capable of rainwater harvesting where water would be purified, filtered, used, and recycled. The skin could even absorb moisture from the air
^43^	Mechanism	The Heliotropic project investigates different skins patterns for the solar shading of existing buildings and proposes a new passive-active surface	smart adaptivity mixing lightweight fabrics and bimetals which harnesses solar energy instead of active, mechanical systems that require more electricity
^32^	Mechanism and Motion	Using the shape-memory alloy (SMA) for climate-adaptive responsive architecture by using 3D-printed attachable kinetic shading device	Self-shaping and HMI intervention allowed (switchable to a motor-gear system controlled by a mobile app)

**Figure 3:**
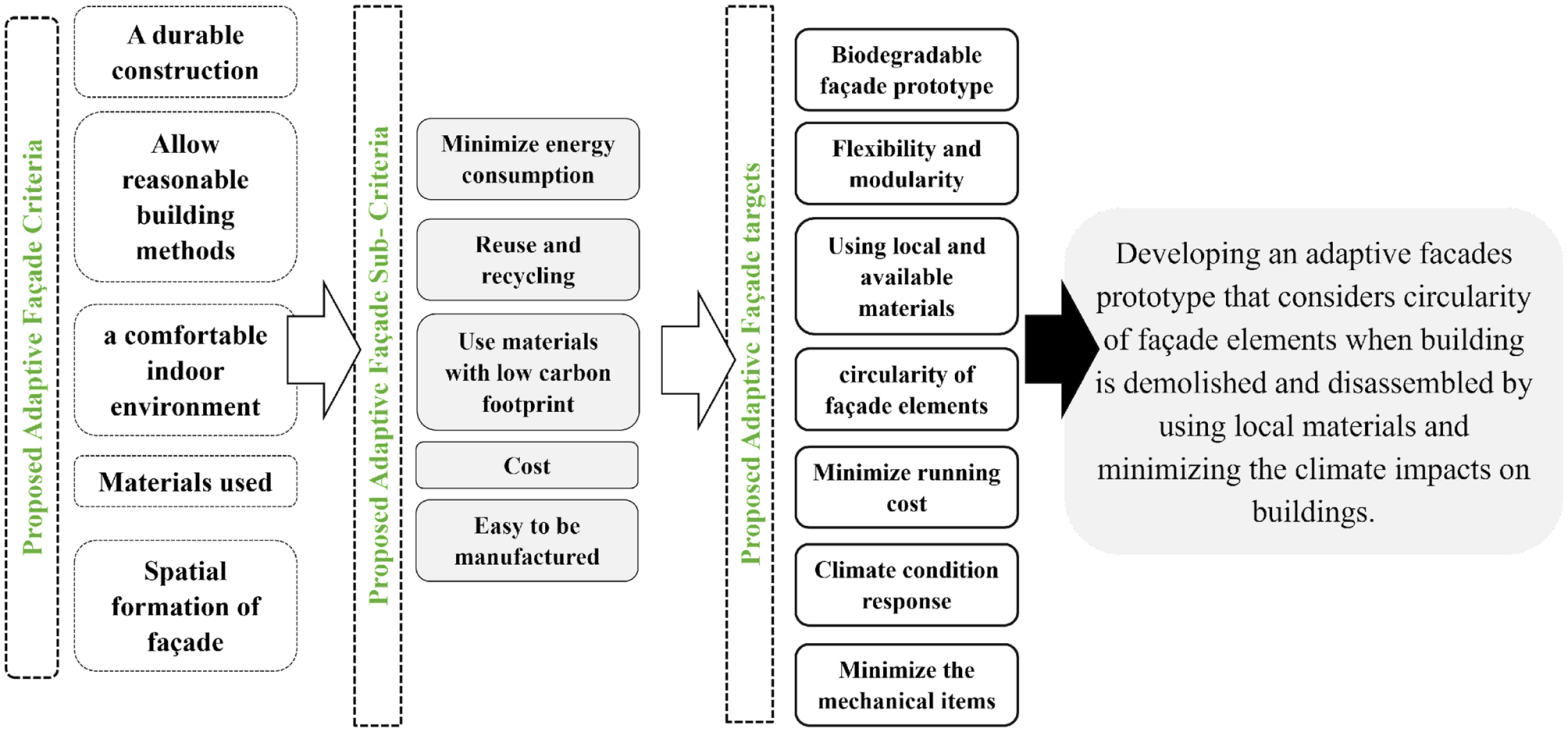
Proposed adaptive façade outcomes and targets by defining the criteria and sub-criteria points from literature in order to formulate the significance items of the proposed module, source by the authors.

Based on the analysis of literature, different criteria and sub criteria points were selected to enhance the workflow of this prototype. Subsequently, those points are addressed such as façade design, durability, mechanism, materials used, building methods, and the impact on the indoor environment. The following step is to adapt the significance of the façade on the building construction. Some key sub-criteria are selected as minimizing the energy consumption, cost, reuse and recycling the construction, use of biodegradable materials with low pollution effects, and ease of manufacturing by local materials. Figure 3 illustrates the sequence of the outcomes for the adaptive façade and some selected parameters as well. This step aims to enhance the selection process of the proposed adaptive module for local climate based on suitable attributes to investigate with locally available materials, while considering the availability, flexibility, modularity, and cost aspects.

## Methodology, material, design and methods

### Research methodology

This work is based on a quantitative empirical study that integrates experiments and tests. It is based on four phases which could be summarized as follows: *Step one:* selection, and modeling phase; A structured literature review analysis which will include reviewing different study cases to define the latent variables actions and systems. Computational modeling using Fusion 360, Rhino software, and the grasshopper plugin with the varying climatic parameters in Cairo as a pilot climate zone. *Step two:* Constructing the up-scale model phase; This phase merges between different fabrication methods for different parts. Using laser cutting to construct half rings and revolving plate parts of the spherical joint from the metal sheet. Conventional manufacturing methods are used for finalizing all parts; drilling and saw blade. Finally, manual assembly is performed. The project encompasses hybrid material classes merging biodegradable and non-biodegradable materials. Local materials are used. Biodegradable materials used were natural jute fiber and beech wood. Jute fiber is used for the shade, while beech wood is used for the support. Non-biodegradable but durable material used is iron Grade 37, for mechanical loading requirements. Iron sheets are used for the spherical joint and fixation support joints.

*Step three:* the sensing and processing phase (*Detection of the input Variables from Real/Field Case*) identify the selected parameters and install hardware and middleware acquisition modules of the model. Smart control is programmed using a Mega Arduino microcontroller. The project includes different components such as motion sensors for occupants’ presence as PIR sensors, temperature sensors such as LDR sensors, servo motors, LED, and resistors. This step will be based on sensing the selected input data variables from a real upscale model. Subsequently, *step four* is the testing phase.

### Design development of the module

**Figure 4:**
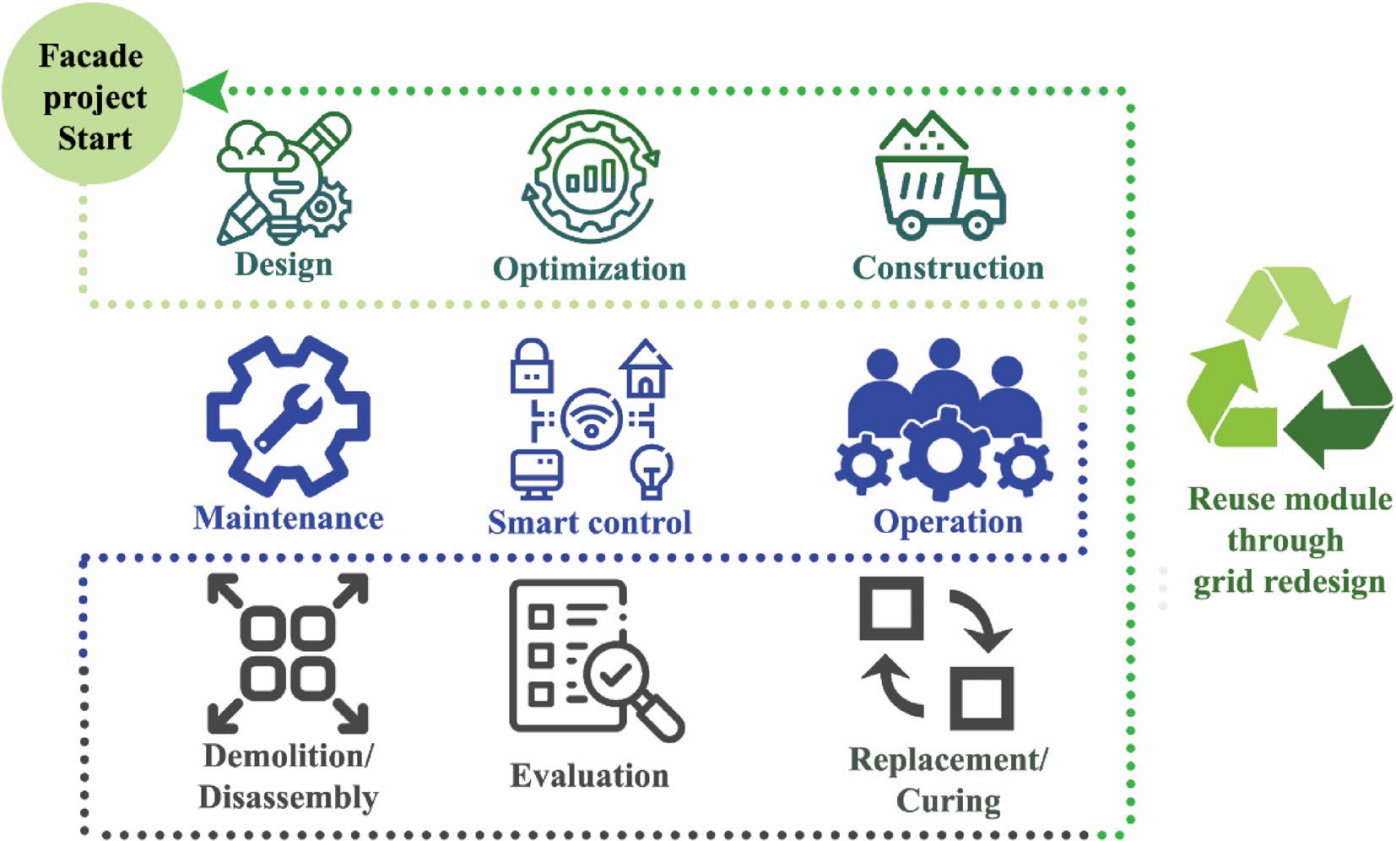
Graphical presentation of proposed adaptive system life cycle. The cycle started by designing the universal adaptive façade module, optimization of material usage and minimizing the actuators, followed by construction and fabrication of the parts. During operation, the façade will moderate the micro-climate to reduce electromechanical loads, with continuous maintenance for motors and the flexible jute textile. The disassembly instead of demolition phase will be done through the replacement and curing of any of the parts, then finally the module offers a flexible adaptive design which can be reused in other buildings. Source by the authors.

**Figure 5:**
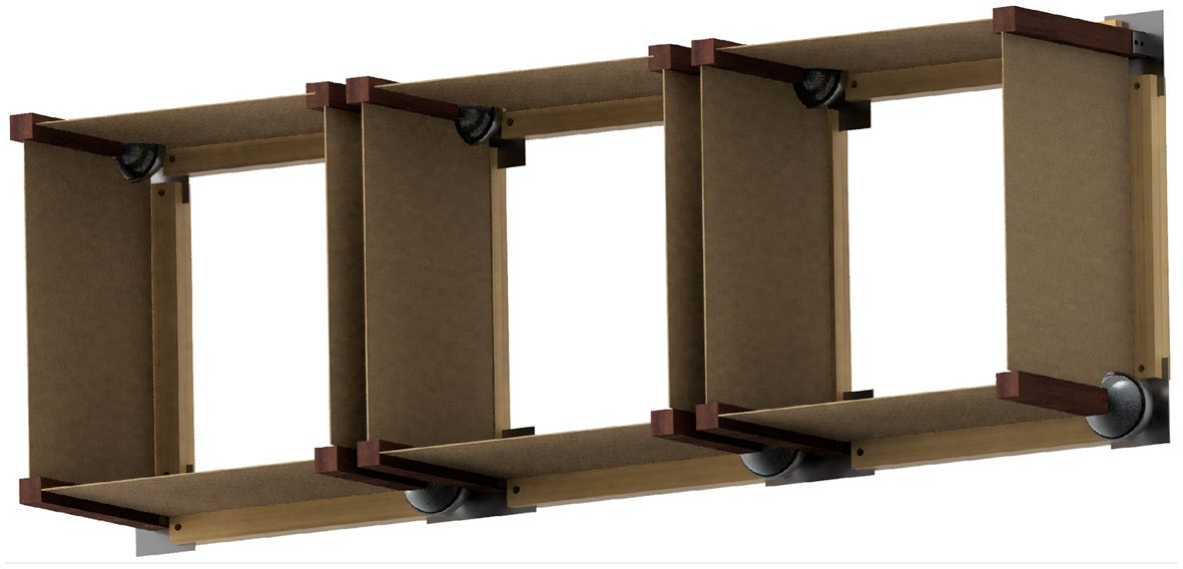
A Graphical computational model of the 3-module proposed adaptive façade, each consisting of 2 fixed and 2 spherical joints. Source by the authors.

Adaptive façade components life cycle as shown in Fig. 4 can be addressed in design phase, through designing the unit for adaptive reuse post demolition or disassembly. Designing for reassembly is one the objectives of this empirical work. An adaptive joint allowing diverse motion iterations is used, i.e. motion during operation and when reassembled in the newly built environment setting. The spherical motion flexibility offered allows diverse shading iterations according to solar intensity level surrounding the different buildings through a smart control. During reassembly, the system would require simple joint material performance evaluation, and minor parts’ treatments if needed. While the sensors will make smart adaptations to the new setting. This will allow enhanced sustainability by exploiting non-biodegradable materials while offering a durable system. Biodegradability is presented in sticks, supports between joints, and natural fiber textiles. The project consists of 3 architectural circular adaptive façade modules. The adaptive façade module proposed consists of 2 fixed and 2 spherical joints as shown in Fig. 5. The Jute fiber textile is planned to be implemented between each two sticks, formulating the semi-translucent shade.

#### Modeling phase (module design)

The circular architecture module unit design concept is to utilize the universal spherical joint motion, providing different possible shading iterations. 180° motion range of movable joint is required across two axes of motion. Design for reassembly is a key objective for the proposed façade system. Partial motion is to be provided in each variable iteration across the operation and redesigned for re-use, according to the motion range needed. During operation, the unit will vary its opening to accommodate occupants’ light and thermal comfort, with the varying sun path across the day, month, and year. Whilst for the design for reuse, the universal spherical motion will allow variable façade design motion range iterations as shown in Table 2.

**Table 2:**
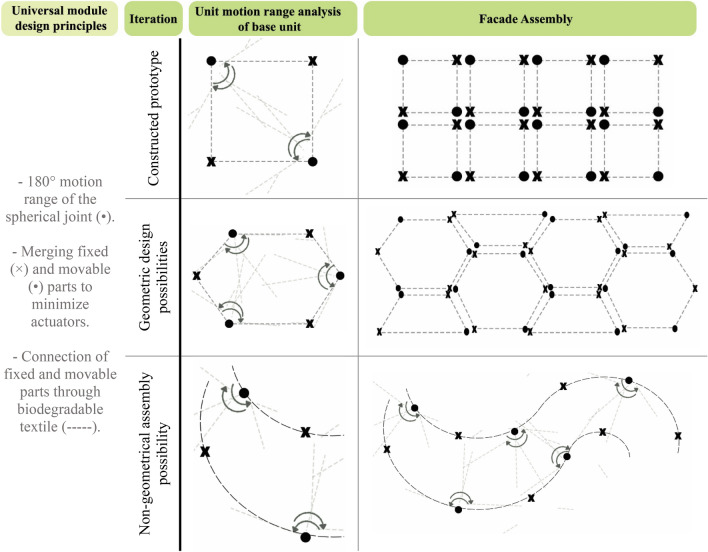
Illustrating the redesign for reuse possibilities.

#### Motion joint

The goal is to design a universal architecture module using a joint that allows circular motion. Two degrees of motion freedom is required which allow adaptive directional motion control to the circular system upon re-use in new building with a different orientation/setting. Thus, facilitating easy reuse with software control update – if needed – and minimum components change.

**Figure 6:**
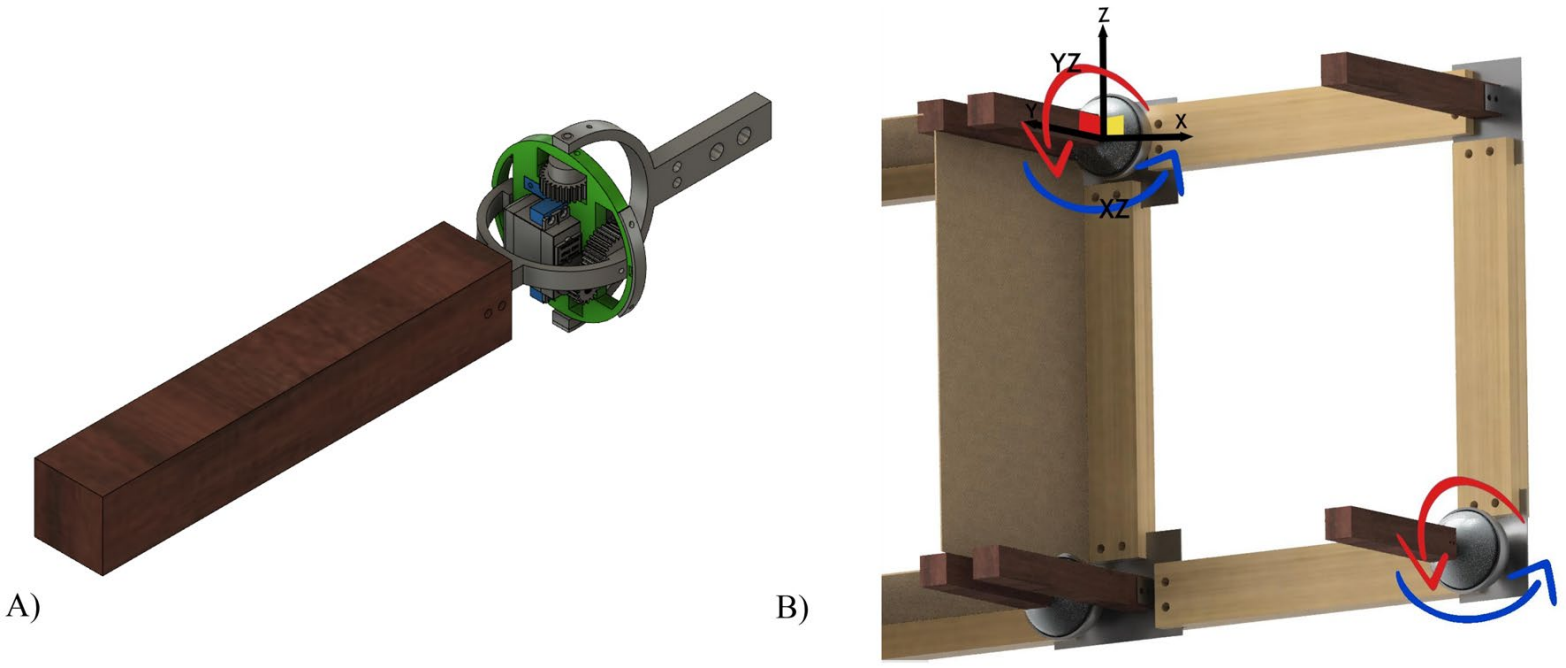
(**A**) spherical joint components. (**B**) schematic degrees of motion of spherical joint.

Robotic spherical joints were found in literature allowing two degrees of motion. In literature^44–48^, several reconfigurable kinematic mechanisms were investigated to provide spherical motion. The selection of the base concept spherical joint design for the façade module depends on two main objectives, which are a minimum number of actuators and minimum volumetric space utilization. The objectives target minimizing energy consumption for actuation and aesthetics. Among the literature, the design described in^49^ fulfills the objectives. The spherical joint of the façade is designed to be a special case of the selected robotic spherical joint design. The joint allows 2-degrees of motion freedom as shown in Fig. 6.

#### Prototype control system logic

The electric system control is designed to adapt to external and internal parameters. An automatic control is established using Mega Arduino micro-controller. The automatic mode begins with data from users’ motion sensors, interior light sensors, and sun sensor as system input. The processor evaluates the outdoor and indoor light intensities, leading to adjusting universal joints of the module angles to optimize internal daylighting with minimum glare. The minimum glare allowed is in specific locations in the room away from work planes. Thus, the module will differ in its opening angle according to the presence of the occupants and climate conditions. Subsequently, indoor artificial lighting is integrated for optimized space lighting throughout the day. There are different hardware used in this model such as Mega Arduino uno as the brain for the whole structure, PIR (passive infrared sensor) motion sensor as the occupant detection device, LDR (light dependent resistor) sensor for detecting the lighting intensity, and LED (light-emitting diode) for lighting control. For the software programming part in this model, Arduino Ide is used in building the code for this project.

**Figure 7:**
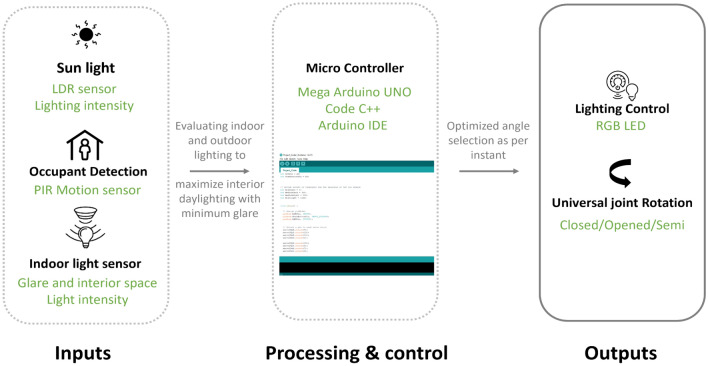
The proposed adaptive façade prototype code logic, source: by the authors.

The module mechanism encompasses a rod with a fixed or rotating spherical joint. The rotating spherical joint enables the rods to rotate at different angles. It can be with three states almost closed, fully opened, and intermediate state. It will interact with the different moods and activities of users in their space which allows the space to be more connected and visible. The rods input motion signals from people detection by using a PIR motion sensor. Then according to the LDR sensor which measures the intensity of the outdoor ultraviolet light rays, the rods move at different angles. In addition, this affects the opening/closure of the LED lights to control the lighting consumption as shown in Fig. 7.

Shedding the light to the programming code for the proposed module. First of all, we define Servo motor library in the Arduino code. As discussed above there are 3 pairs of servo motors (*6 servo motors*) in the whole model which are responsible for the joint motion mechanism in the proposed adaptive modules. Then we define the opening and closing angles which are responsible for calculating the most suitable angle degree according to the outdoor environment status (*intensity of the outdoor ultraviolet light rays and occupants’ detection*). Angles can be in three states almost closed, fully opened, intermediate state. The base state angle degree is 0 and it changes with the outdoor light intensity and occupant’s motion. Then we define the LDR (*light dependent resistor*) sensor and indoor LED sensor with defining threshold points for LDR sensor readings. From climate studio and weather files lighting threshold averages were defined as MostDark, MediumDark, MediumLight, MostLight points. Subsequently, servo motors take input motion signals from people detection by using a PIR motion sensor. Then according to the LDR sensor which measures the intensity of the outdoor ultraviolet light rays, servo motors move at different angles. So, we define different cases for 3 module states in order to differ their opening angle according to the presence of the occupants and outdoor climate conditions (*outdoor lighting intensity*).

## Results and discussion

### Design and demonstration

**Figure 8:**
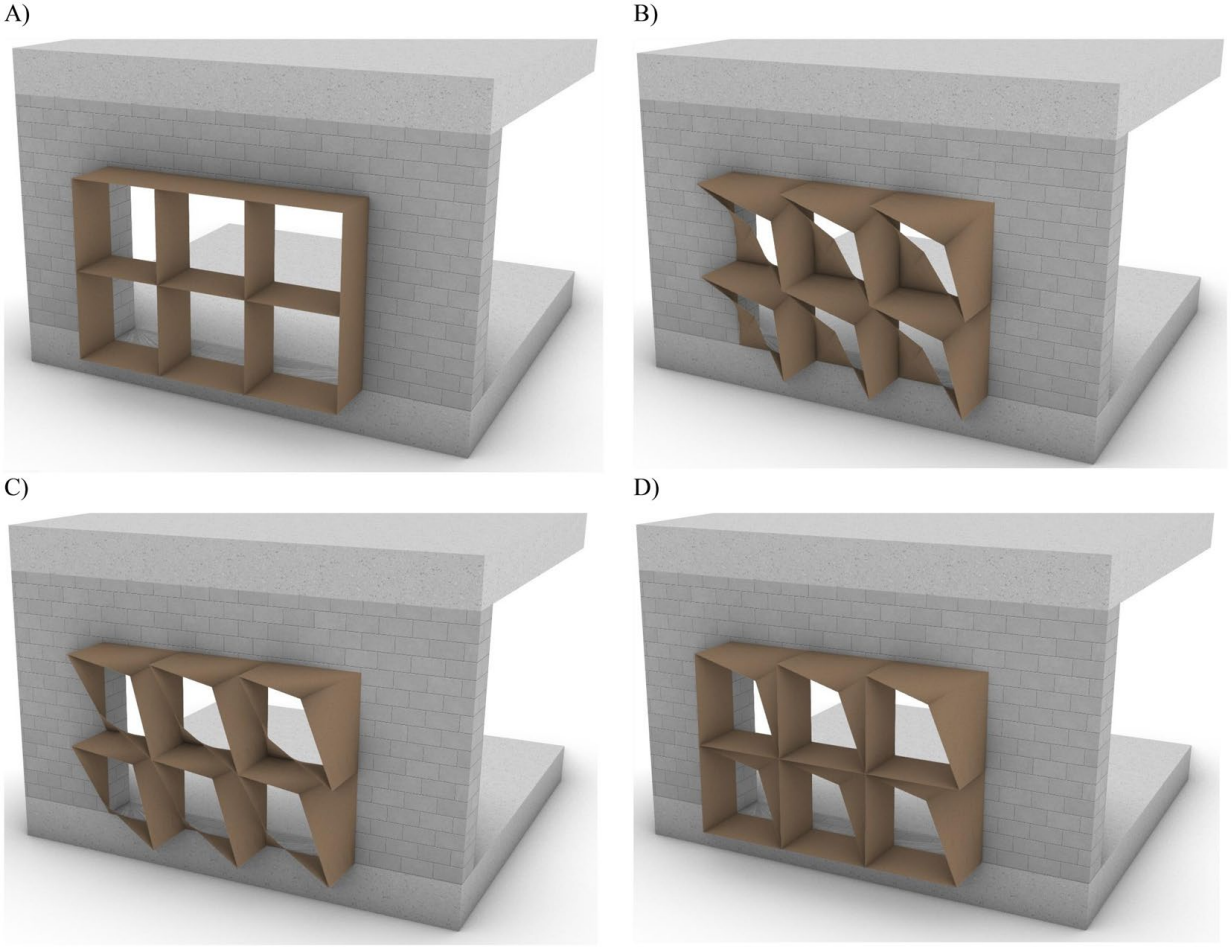
Basic circular adaptive module iterations corresponding to light and thermal comfort required by occupants. (**A**) Fully opened state. (**B**) Almost closed state (**C**,**D**) Partially closed state. Source by authors.

A circular biodegradable adaptive façade system module is constructed for optimized energy efficiency and performance of buildings in Egypt. The project utilized locally available materials for production. The different produced circular façade states are described as follows; *Maximum/full opening* state for detecting someone in space with a low outdoor lighting intensity. The *almost closed/minimum opening* state occurs when there is direct solar radiation on the module is detected. The *intermediate state* occurs when detecting an occupant with partially directed solar radiation on the circular module. So, it will balance visual connectivity and thermal comfort for occupants. Subsequently, the adaptive façade will emit different indoor light cases according to its opening angle states as shown in Fig. 8.

**Figure 9:**
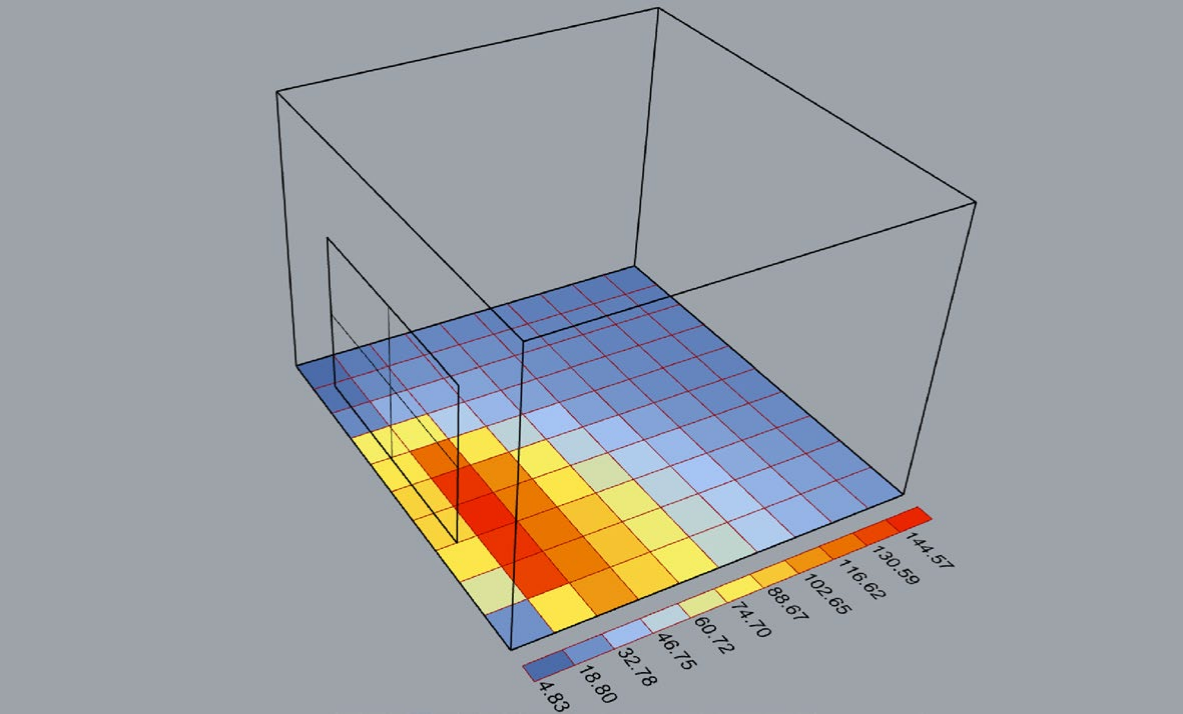
Masses abstraction for honeybee and Ladybug tools components visualization.

In Table 3, Energy and lighting computational simulations are conducted using Ladybug and Honeybee components in grasshopper plugin in Rhinoceros software, which uses OpenStudio and EnergyPlus engines by linking Rhino CAD environment with them^50^. Most electromechanical loads increase after 12 PM in all building typologies in Egypt, to overcome the glare and thermal discomfort in interior building spaces. This corresponds with activity time duration in Egypt. Thus, the west, south-west, and south façades are chosen above others for the simulation due to Egypt’s location in the northern hemisphere. Helwan weather was chosen due to the EPW weather file and diverse building typology availability. Spatial abstraction is executed for simulation requirements as shown in Fig. 9.

**Table 3:** Ladybug and honeybee simulations results.

Orientation	Opening state	GA (%) “Top view”	Cumulative radiation (kWh/m^2^) over the weather time
West	No circular module		
	Almost closed/minimum opening state		
South-west	No circular module		
	Intermediate		
South	No circular module		
	Maximum/full opening state

**Figure 10:**
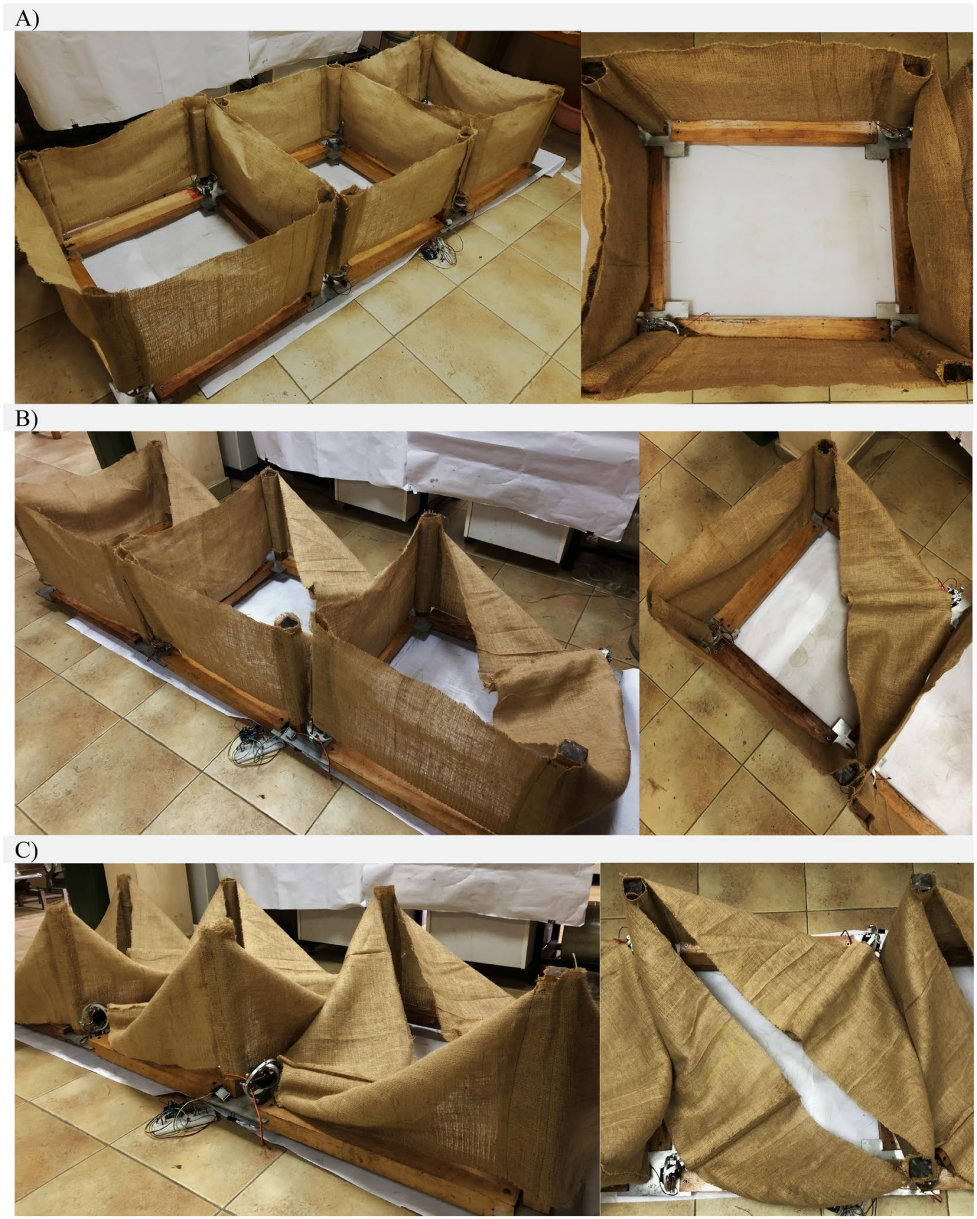
Fabricated prototype of the proposed adaptive modules and experiments. Each expected module state is presented across a single module, and the fabricated 3- module cluster. (**A**) The module is in fully open state when no shading is needed. (**B**) Intermediate state when partial shading is needed. (**C**) Almost closed state when maximum low-level sunrays are found.

Basic office occupancy schedule is defined. The floor is divided into a grid of 100 sensor points. Each shading state simulation is executed separately. Honeybee is used to run annual glare study resulting in glare autonomy (GA) and calculating the cumulative radiation. Ladybug is used for data visualization. All results are shown from the floor plan view. The textile is abstracted with no natural deformation. Thus, the lighting simulations are a representation of the idea, while the smart Arduino control system will compensate for the deviations in this simulation because of the indoor light intensity sensors. It is evident from the results that the façade adapts when solar radiation penetration reduction is required. Thus, less glare and thermal discomfort are found, while maintaining the visual contact with outside the building after the direct radiation is not found. Subsequently, the following Fig. 10 presents the real live pictures for the proposed adaptive modules and lab experiments. As our first phase of this research, the modules were placed on a flat surface and then we performed our trials and tests as discussed above. Each state was recorded and captured during our trials.

### Adaptive façade module and human centered design

The proposed adaptive façade module can create a reciprocal relation between users and space to make our spaces more adaptable and to be used with different functions. Table 4 illustrates the outcomes of the adaptive façade across different sections. According to the control/operation, the façade shows adaptability through different iterations in correspondence to the climate condition and users’ detection. The circular adaptive module considers flexibility and modularity, level of visibility, and functionality level. In addition, it can raise the satisfaction level and indoor comfort for users. Finally, the material biodegradability type, circularity, and long-term financial cost.

**Table 4:** A framework of the proposed module with the achieved targets and outcomes.

Criteria	Sub-criteria	Illustration
Control/operation	Adaptability of the system	The movable rod of the façade prototype changes its angles as different iterations through the outdoor temperature and users’ detection
	Type of trigger (INPUT)	Outdoor temperature and users’ detection
Design	Flexibility and modularity	The module of this façade could be adapted and repeated according to the wall design and location
	Level of visibility	It ranges from full visibility to partial visibility
	Functionality level	It is related to the climate, indoor comfort level, and users’ preferences
End user	Satisfaction level and indoor comfort	The adaptability of this façade prototype can raise the satisfaction level of users because it could control the indoor temperature and the lighting level as well
Materials	Biodegradable	The façade prototype natural materials
	Non-biodegradable	Designed for continuous reuse for enhancing life cycle
	Type of actuators	Smart electronics such as PIR motion sensors for the occupant detection device, Servo motors for façade control, LDR for lighting intensity, and LED for lighting control
	Material environmental effects	Minimizing waste pollution
Economy	Operation cost	It affects the indoor lighting consumption and the indoor comfort level of users. By default, this proof of concept can mitigate the climate impacts by minimizing energy consumption and raising the visual quality of the space
	Reuse (circularity)	Parts of this prototype could be re-used and adapted into different functions

The significance of this project is to enhance the circularity and modularity criteria of adaptive façades. In comparison with previous research investigations, this adaptive module offers unique circular adaptability across design, operation, and reuse phases. The adaptability is found through the building life cycle, after demolition, and possible adaptive redesign of the same building for energy or aesthetic requirements. Much research has introduced the idea of kinetic automated facades, but this proposed module has a unique idea of how the state of openings could be changed whenever the climate changes. The spherical joint is designed to rotate along two axes of motion depending on the smart control. It is clear that buildings will face changes throughout their life span under the circumstances of climate change. There is no fixed state of our buildings from now on. Another unique feature of the module is presented, which is the ease of adjusting the prototype materials according to local availability in the region. Under the circumstances of climate change, this idea could help in minimizing the challenges of climates to be adaptable, flexible, and reusable.

In future investigations, universal spherical joint allocation optimization analysis should be studied to provide nongeometrical iterations with minimum actuators allocated, while catering to occupants and energy consumption requirements. From our investigations, we should propose that another durable motor is needed to better enhance the spherical joint output effects and to minimize the annual maintenance for this module as well. Diverse natural local biodegradable materials like rice straw, natural loofah and flax fibers can be investigated as possible replacement for wooden supports and the shade. Palm trees midribs can be investigated as adaptive façade support parts, as they are proven to be of strong structural reliability^51^. Using recycled materials like plastics in fabricating the spherical joint can be investigated to provide a sustainable inclusion of plastic waste in a re-usable part. Further investigation is needed to explore biodegradable stiffeners along each flexible jute textile part, which could provide diverse morphologies according to owners’ preferences and space function requirements. From our observations, it could be enhanced by adding a wooden grid between each rod to fix more jute mesh.

## Conclusion and future work

Integrating circularity in adaptive façades will help enhance the building envelope component value. A circular adaptive façade module is constructed in this study as proof of concept. Subsequently, this proposed module could promote a different approach to ensure the interactions between buildings, climate change, and subsequent potential effects on building occupants, energy efficiency, and the built environment. The module offers three levels of adaptability. It will encourage decision-takers, especially building owners, to install it as it can be resold, thus keeping its value while minimizing building operation running costs. It is designed to minimize the electromechanical loads by investigating the optimum material and motion assembly selection and optimizing the adaptive façade life cycle while reducing waste of manufacturing and costs. Prefabricated biodegradable elements have been suggested to fill the gap of circularity and waste in materials. This research project depends on the wide-scale adoption of new building technologies to bring down prices and make them affordable to find alternative ways. It adds the adaptability feature to building façades through local elements. Which are feasible due to the availability and relatively cheaper electronic elements.

The proposed adaptive module can improve and, in many cases, drastically reduce energy consumption. It can be produced in different building types located in various climate zones. It utilizes different local materials and smart controls. The controllable operation system also provides occupants with options for their personalized indoor comfort, improving user satisfaction. It considers the most suitable climatic adaptation for each location and achieves component combinations that satisfy sets of project requirements and indicators. This study is based on the circularity gap between established adaptive façade research directions in architecture. The focus of this study is based on some selected utilities such as indoor lighting, and outdoor lighting intensity in the building. The flexibility and modularity criteria of this module could be used and enhanced in different regions by adjusting the materials according to the local product in each region. Thus, it can be adapted to various regions with long solar radiation hours as a general global solution.

Module physical customization and installation on an office window is a part of future research to verify and adapt this idea along an existing building façade. Different iterations and empirical studies are expected. Furthermore, some investigations will be conducted on the material's mechanical properties, and the surface deflection of textile studies due to gravity. As a future work, a focus on the material's embodied energy and life cycle analysis of the module design is expected. It will focus on the process thus implying the identification of materials and parameter inputs. Various illustrations and discussions were elaborated by researchers to analyze the embodied energy^52^. Future work will depend on the analysis development of a consistent and comparable database of the parameters identification. Then, the proposed adaptive module will be evaluated according to three dimensions cost, environmental, and social concerns. Through life cycle assessment there is a possibility to analyze and improve the sustainability of proposed module, services, and processes. Different scientific methodologies and life cycle strategies were introduced as in^53,54^, which if merged with the presented module, can be produced as an industrial product for implementation in the construction industry. This module can be introduced to the market to attract business sponsors and produce a local product. It is possible to develop from other interdisciplinary views and further upcoming new techniques and industrial applications in the future.

## Data Availability

The datasets generated during and/or analyzed during the current study are available from the corresponding author upon reasonable request.
